# Periodontal disease in preoperative patients with digestive cancer: a retrospective, single-institution experience in Fukui, Japan

**DOI:** 10.1186/s12903-020-01378-y

**Published:** 2021-01-06

**Authors:** Shinpei Matsuda, Takanori Goi, Yoshio Yoshida, Hitoshi Yoshimura

**Affiliations:** 1 Department of Dentistry and Oral Surgery, Unit of Sensory and Locomotor Medicine, Division of Medicine, Faculty of Medical Sciences, University of Fukui, 23-3 Matsuokashimoaizuki, Eiheiji-cho, Yoshida-gun, Fukui, 910-1193 Japan; 2 First Department of Surgery, Faculty of Medical Sciences, University of Fukui, Fukui, Japan; 3 Department of Obstetrics and Gynecology, Faculty of Medical Sciences, University of Fukui, Fukui, Japan; 4 Center for Preoperative Assessment, University of Fukui Hospital, Fukui, Japan

**Keywords:** Periodontal disease, Caries, Digestive malignant disease, Surgery, General anesthesia

## Abstract

**Background:**

The careful preoperative oral assessment may be useful for safe surgery under general anesthesia. The purpose of this study was to investigate the presence of periodontitis in patients with malignant digestive disease before surgery under general anesthesia.

**Methods:**

Patients with digestive malignant disease who underwent periodontal examination and orthopantomograph examination for preoperative oral health assessment were participated. The authors investigated the patients’ general characteristics and clinical oral information, including the presence of periodontitis.

**Results:**

One hundred twenty patients participated in this study. The mean and standard deviation of the number of teeth was 20.8 ± 8.2, and there was a statistically significant correlation between age and number of teeth. The periodontal pocket depth was 3.0 ± 1.0, and mobile teeth were observed in 62 patients. There was a statistically significant correlation between number of teeth and number of mobile teeth. However, there was no significant difference between the age of patients without mobile teeth and the age of patients with mobile teeth.

**Conclusions:**

This retrospective study performed in single-institution clarified the presence of periodontitis in patients with malignant digestive disease before surgery. Regardless of age, it is important to assess the oral health, including periodontitis, for safe surgery under general anesthesia.

## Background

Infectious complications, such as ventilator-associated pneumonia (VAP) and surgical site infection (SSI), occur in hospitalized patients and lead to serious conditions [1–3]. They also could cause social and economic disadvantages, including subsequent complications. Previously, some literature reported that oral care could reduce postoperative complications such as VAP and SSI, and medicoeconomic costs were cut at the same time [1, 2, 4, 5]. In Japan, the government has recommended oral care associated with surgery or/and chemoradiation therapy since 2012 to prevent postoperative complications and reduce side-effects [4]. As a result, there have been many reports, mainly from Japanese researchers, regarding the effects of preoperative oral and dental health management for prevention of postoperative complications [2, 4–6]. Furthermore, some recent literature has shown interesting information about the association between oral microbiota and malignant digestive diseases [7, 8].

Periodontal disease, including gingivitis and periodontitis, is a chronic inflammatory disease induced by substances in the microbial plaque that affects the gums and the alveolar bone, and it leads to tooth mobility and loss [9–11]. It is highly prevalent, and disease severity increases with age [10, 11]. Some studies suggest an association between periodontal disease and some systemic diseases such as diabetes mellitus, cardiovascular disease [9]. Our previous study about tongue cleaning prevalence clarified that the necessity for education about oral care is widespread not only for the general public at various ages but also among medical professionals [3]. However, that study was a questionnaire survey and there was no additional information about the clinical oral environment and periodontal disease [3].

In recent years, there is growing evidence associating different mechanistic links between periodontitis and some malignant digestive diseases, as a noteworthy topic [8, 10]. It is an important research field in order to provide oral health education to the general public.

The purpose of this retrospective study was to investigate the presence of periodontitis in patients with malignant digestive disease, before surgery under general anesthesia.

## Methods

### Ethical and study design

Patients with malignant digestive disease who underwent dental assessment at the Department of Dentistry and Oral Surgery at the University of Fukui Hospital between September 2018 and August 2019, and who underwent periodontal examination and orthopantomograph examination for preoperative oral health assessment for general anesthesia, participated in this study. Patients with various stage of periodontitis and periodontal conditions were included, and edentulous patients were excluded from this study [12]. The examiner was one dental hygienist who had had over 5 years of clinical experience, and belong to the author’s department. Periodontal pocket depth was examined using stainless periodontal pocket prove. The authors did not inform the examiner that the authors would conduct the one year retrospective study in order to rule out the biases of examiner. Therefore, it had been not possible to calculate and adjust the sample size to address the hypothesis of this study. This study was approved by the Institutional Research Board (Ethical Committee of University of Fukui, Faculty of Medical Sciences; No. 20190082). Verbal informed consent was obtained from all participants at the time of dental examinations at the Department of Dentistry and Oral Surgery at the University of Fukui Hospital. Ethical Committee of University of Fukui, Faculty of Medical Sciences approved this procedure because the information of this retrospective study have been released to the public.

### Data extraction

The authors investigated the patients’ general characteristics as follows: (1) gender, (2) age, and (3) organ of disease scheduled for surgery under general anesthesia. In addition, for investigation the presence of periodontitis, the authors also extracted those patients’ clinical oral information as follows: (4) number of teeth, (5) number of teeth, excluding residual tooth roots, (6) periodontal pocket depth, (7) number and sites of mobile teeth and degree of tooth mobility, and (8) dental management of mobile teeth. Impacted teeth only observed by orthopantomograph examination were excluded from this study, and residual tooth roots were excluded from measurement of periodontal pocket depth. Tooth mobility was classified by Miller’s classification [11].

### Statistical analysis

The relationships between age and number of teeth, age and number of mobile teeth, and number of teeth and number of mobile teeth were analyzed statistically by Spearman’s rank correlation coefficient. In addition, the age of patients without mobile teeth and the age of patients with mobile teeth were analyzed by Mann–Whitney’s *U*-test. Statistical analyses were performed using IBM SPSS Statistics 25 statistical software (IBM, Tokyo, Japan). *P* < 0.05 was considered statistically significant.

## Results

In this study, 120 patients with various digestive cancers participated . The patients consisted of 82 males (68.3%) and 38 females (31.7%) (Table 1). The mean age and standard deviation of those patients were 68.2 ± 11.2 years. The youngest patient was 40 years old, and the oldest patient was 91 years old. The most common organ of disease scheduled for surgery under general anesthesia was the colon (55 patients, 45.8%), the second was the stomach (31 patients, 25.8%), and the third was the pancreas (11 patients, 9.2%).

**Table 1:** The general characteristics of patients

	Cases (n)	(%)
Gender		
Male	82	68.3
Female	38	31.7
Total	120	100
Age		
≦59	24	20
60–79	75	62.5
80≦	21	17.5
The digestive organ localizing malignant disease		
The colon	55	45.8
The stomach	31	25.8
The pancreas	11	9.2
The esophageal	8	6.7
The liver	8	6.7
Others	7	5.8
The presence or absence of mobile teeth		
Presence	62	51.7
Absence	58	48.3

**Fig. 1:**
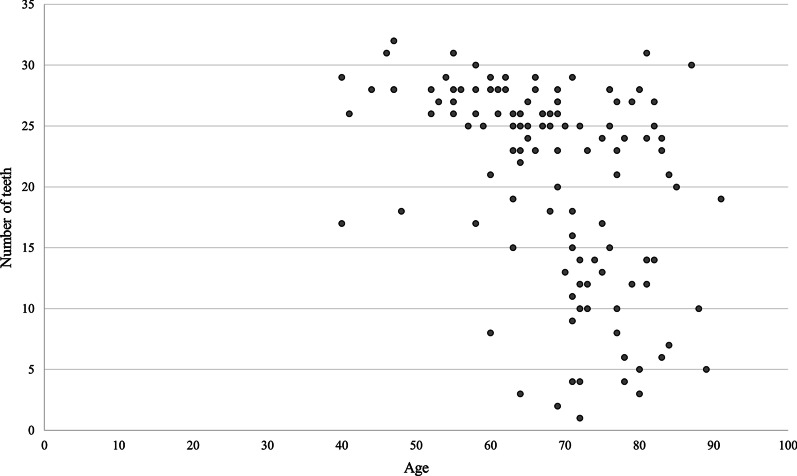
Age and number of teeth

**Fig. 2:**
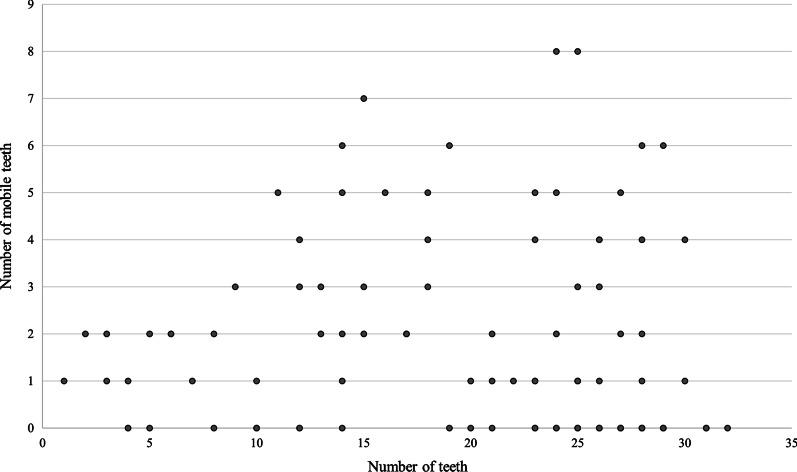
Number of teeth and number of mobile teeth

Regarding the number of teeth, the mean and standard deviation was 20.8 ± 8.2, and the number of teeth, excluding residual tooth roots, was 20.2 ± 8.5. Residual tooth roots were observed in 14 patients (11.7%). The maximum case was 32 teeth, and the least case was only one tooth. There was a statistically significant correlation between age and number of teeth (*P* < 0.05, Spearman’s rank correlation coefficient) (Fig. 1). The mean and standard deviation of periodontal pocket depth was 3.0 ± 1.0. Mobile teeth were observed in 62 patients (51.7%), and the mean number of mobile teeth and standard deviation in those 62 patients was 2.9 ± 1.9. The mean and standard deviation of degree of tooth mobility was 1.3 ± 0.5. The mean age and standard deviation of age of the patients without mobile teeth was 67.3 ± 12.1, and the mean age and standard deviation of age with mobile teeth was 69.0 ± 10.2. There was no significant difference between the age of patients without mobile teeth and the age of patients with mobile teeth (*P* = 0.42, Mann–Whitney’s *U*-test). There was no statistically significant correlation between age and number of mobile teeth (*P* = 0.76, Spearman’s rank correlation coefficient), and there was a statistically significant correlation between the number of teeth and the number of mobile teeth (*P* < 0.05, Spearman’s rank correlation coefficient) (Fig. 2). Maxillary mobile teeth were observed in 49 patients, and mandibular mobile teeth were observed in 39 patients. The most common site was the right maxillary molar region (22 patients, 35.5%); the second was the left maxillary incisor region (20 patients, 32.3%).

For evaluation of the association between digestive organs with malignant diseases and periodontitis, the patients who participated in this study were divided into two groups: patients with malignant colorectal disease (55 patients) and the other patients (65 patients). Regarding the number of teeth, excluding residual tooth roots, the mean and standard deviation of the patients with malignant colorectal disease was 20.3 ± 8.4, and that of the other patients’ group was 20.2 ± 8.5. In addition, there was no significant difference between the number of teeth, excluding residual tooth roots of the patients with colorectal malignant disease group, and that of the other patients’ group (*P* = 0.93, Mann–Whitney’s *U*-test). Among the patients with malignant colorectal disease, the mean and standard deviation of periodontal pocket depth was 3.1 ± 1.0, and mobile teeth were observed in 33 patients (50.8%). In the other patients’ group, the mean and standard deviation of periodontal pocket depth was 3.0 ± 0.9, and mobile teeth were observed in 29 patients (52.7%). There was no significant difference between the periodontal pocket depth of patients with malignant colorectal disease and that of the other patients’ group (*P* = 0.96, Mann–Whitney’s *U*-test).

As dental management of mobile teeth, mouth guards were made for 16 patients, and tooth extractions were performed in six patients, before surgery under general anesthesia.

## Discussions

This study clarified that there was no significant difference between the periodontal pocket depth of patients with malignant colorectal disease and that of the other patients’ group.

This study was conducted in Fukui Prefecture, Japan; it reports that about the half of the preoperative patients with malignant disease had mobile teeth. There was a statistically significant correlation between age and number of teeth; however, there was no statistically significant correlation between age and number of mobile teeth. In addition, there was a statistically significant correlation between number of teeth and number of mobile teeth but no significant difference between the age of patients without mobile teeth and the age of patients with mobile teeth. These results suggested that age cannot be a predictor of the presence or absence of mobile teeth and that careful preoperative oral assessment is important for safe surgery under general anesthesia with endotracheal intubation, regardless of age.

Although the authors considered that their previous study about tongue-cleaning habits had shown increasing interest in oral care among the general public at various ages, this study clarified that those results did not necessarily reflect the actual oral health conditions, such as periodontal and caries management [3]. Regarding the divergence of these results obtained by studies performed in same target area, namely the increasing interest in oral care and little interest in periodontal disease, we had to take into account the difference in the ages of the participants between those studies. On the other hand, we thought this divergence may be related to the difference between the participants with malignant digestive diseases scheduled for surgery and the participants without it. In this study, there was no significant difference between periodontal pocket depth in the group of patients with colorectal malignant disease and that of the other patients’ group. Furthermore, the number of teeth, excluding residual tooth roots, was not significantly different between the group of patients with malignant colorectal disease and periodontal pocket depth of the other group of patients. For a discussion about the association between oral health and malignant diseases, the authors will have to conduct a larger study and population-based studies targeting the same patient groups on various aspects of those factors in the future.

Tooth mobility is an important risk factor for endotracheal intubation, and has been discussed in much of the literature, to perform safe surgery under general anesthesia [13–17]. Gaiser et al. reported that dental trauma was the most common complication of general anesthesia, and the incidence of dental injury under general anesthesia, when provided by an anesthesia resident, was 0.1% [14]. In addition, large-scale surveys about peri-anesthetic dental injury have reported that the incidence of such injuries ranges from 0.02 to 0.27%, and 75% of those occurred during intubation maneuvers for elective surgery [15–17]. Giraudon et al. reported that the most common type of dental injury was fracture of crowns and teeth, and the second was tooth avulsion or luxation [17]. A complete evaluation of the oral or dental conditions by an experienced anesthesiologist was recommended [15].

Recently, the establishment of centers for assessment and management of admitted patients, comprising related occupations, such as anesthesiologists, pharmacists, nurses, and so on, is progressing in Japan [18]. The results of the current study suggest that preoperative oral assessment, such as for periodontal disease, for patients with malignant digestive diseases can provide important information for avoiding oral complications during general anesthesia with endotracheal intubation to anesthesiologists. Thus, the authors consider that adding dental professionals to the centers for patient assessment may increase the accuracy of oral assessments and reduce oral complications during surgery under general anesthesia as well as reduce the burden on anesthesiologists. If it is impossible to extract affected teeth, or if surgery is near at hand, dental professionals can suggest making mouth guards when the preoperative patient has a high number of mobile teeth [19]. The authors consider that the management of mobile teeth in such departments, including tooth extraction, making mouth guards, and giving anesthesiologists warnings about mobile teeth, could contribute to preventing dental complications during surgery under general anesthesia.

Numerous studies suggest an association between periodontitis and malignant diseases. Komiya et al. reported that patients with colorectal cancer had identical strains of *Fusobacterium nucleatum*, associated with periodontitis, in their cancer and oral cavity [7]. In addition, *Porphyromonas gingivalis* is an important pathogen that causes periodontitis, and a relationship between it and some malignant digestive diseases, such as esophageal, gastric, hepatocellular, colorectal, and malignant pancreatic diseases, has been considered [8]. Moreover, the maintenance of the oral health environment may contribute to the prevention of systemic disease, including malignant disease, not only dental diseases such as periodontal disease and caries [8]. Further basic and clinical studies are necessary to clarify the effect of periodontal treatment on tumorigenesis.

The authors focused on patient with digestive cancer and performing safe surgery. The limitation of this study was a lack of dental history and periodontal parameters such as bleeding on probing, plaque index, clinical attachment level, and ranges of periodontal sites (e.g. shallow, moderate, and deep). And also, although the authors had understood the importance of evaluation the progression of periodontitis using longitudinal evidence of clinical attachment loss or bone loss described in the consensus report of classification of periodontal and peri-implant diseases and conditions reported by in 2018, periodontal pocket depth evaluated by traditional method was chosen in order to rule out the bias of examiner and the examination error in this study [20]. Additionally, because the authors did not divide the severity of periodontitis and cancer, this study could not analyze straight relationships with the presence of periodontitis and cancer. Further studies are needed in order to suggest the correlation between periodontal parameters and types of cancer, or between stages of periodontitis and stages of cancer. And then, results of those studies may clarify biological condition that would explain the prevalence of periodontal disease among persons with cancer. If future large-scale studies with multi-time examination at different times using new classification of periodontal diseases and conditions suggest the strong relationships between cancer and periodontitis, those results will raise interest in oral health including periodontitis among the general public [20]. In addition, those examinations will provide important information based on new classification to clinicians performed surgery under general anesthesia.

It is undesirable for patients with malignant diseases to be subjected to a delay associated with the presence of dental diseases when starting treatment. We consider that this study may help educate the general public in various age groups about the importance of maintaining good oral and dental health at all times.

## Conclusions

This study clarified the presence of periodontitis in patients with malignant digestive disease before surgery under general anesthesia, on the other hand, there was no significant difference between the periodontal pocket depth of patients with malignant colorectal disease and that of the other patients’ group. The age of patients with malignant diseases cannot be a predictor of the presence of mobile teeth. Regardless of age, it is important to assess the oral health, including for periodontal disease, to ensure safe surgery under general anesthesia. Although this study could not analyze a straight relation with the presence of periodontitis and cancer, an important information had been provided for clinicians involved in cancer treatment by this study.

## Data Availability

The data used to support the findings of this study are available from the corresponding author upon request.

## Abbreviations

VAP: Ventilator-associated pneumonia
SSI: Surgical site infection
